# Complete genome sequence of *Acetohalobium arabaticum* type strain (Z-7288^T^)

**DOI:** 10.4056/sigs.1062906

**Published:** 2010-08-20

**Authors:** Johannes Sikorski, Alla Lapidus, Olga Chertkov, Susan Lucas, Alex Copeland, Tijana Glavina Del Rio, Matt Nolan, Hope Tice, Jan-Fang Cheng, Cliff Han, Evelyne Brambilla, Sam Pitluck, Konstantinos Liolios, Natalia Ivanova, Konstantinos Mavromatis, Natalia Mikhailova, Amrita Pati, David Bruce, Chris Detter, Roxanne Tapia, Lynne Goodwin, Amy Chen, Krishna Palaniappan, Miriam Land, Loren Hauser, Yun-Juan Chang, Cynthia D. Jeffries, Manfred Rohde, Markus Göker, Stefan Spring, Tanja Woyke, James Bristow, Jonathan A. Eisen, Victor Markowitz, Philip Hugenholtz, Nikos C. Kyrpides, Hans-Peter Klenk

**Affiliations:** 1 DSMZ - German Collection of Microorganisms and Cell Cultures GmbH, Braunschweig, Germany; 2 DOE Joint Genome Institute, Walnut Creek, California, USA; 3 Los Alamos National Laboratory, Bioscience Division, Los Alamos, New Mexico, USA; 4 Biological Data Management and Technology Center, Lawrence Berkeley National Laboratory, Berkeley, California, USA; 5 Oak Ridge National Laboratory, Oak Ridge, Tennessee, USA; 6 HZI – Helmholtz Centre for Infection Research, Braunschweig, Germany; 7 University of California Davis Genome Center, Davis, California, USA

**Keywords:** anaerobe, mesophile, halophile, chemolithotroph, methylotroph, organotroph, degradation of betaine, consumption of trimethylamine, homoacetogen, *Clostridia*, *Halanaerobiales*, GEBA

## Abstract

*Acetohalobium arabaticum* Zhilina and Zavarzin 1990 is of special interest because of its physiology and its participation in the anaerobic C_1_-trophic chain in hypersaline environments. This is the first completed genome sequence of the family *Halobacteroidaceae* and only the second genome sequence in the order *Halanaerobiales*. The 2,469,596 bp long genome with its 2,353 protein-coding and 90 RNA genes is a part of the *** G****enomic* *** E****ncyclopedia of* *** B****acteria and* *** A****rchaea * project.

## Introduction

Strain Z-7288^T^ (= DSM 5501 = ATCC 49924) is the type strain of the species *Acetohalobium arabaticum*, which is the type species of the genus *Acetohalobium* [1,2]. The genus name derives from the Latin word ‘acetum’, meaning vinegar, and the Greek words ‘halos’ and ‘bios’, meaning salt and life, respectively, in order to indicate an acetate-producing organism living in salt [3]. The species name derives from Arabat, a peninsula between the Sea of Azov and Sivash [3], since the strain was isolated from lagoons of the Arabat spit (East Crimea) which separates Sivash lake from the Sea of Azov [2]. Currently, this is the only known strain in the genus *Acetohalobium*. *A. arabaticum* participates together with other halophilic bacteria and the genera *Methanohalophilus* and *Methanohalobium* in the C_1_-trophic chain in hypersaline environments [2]. Here we present a summary classification and a set of features for *A. arabaticum* Z-7288^T^, together with the description of the complete genomic sequencing and annotation.

## Classification and features

**Figure 1:**
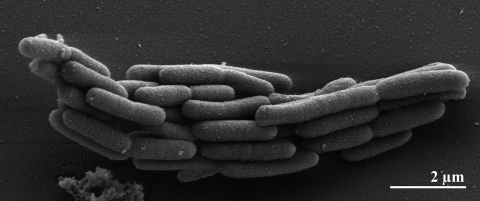
Scanning electron micrograph of *A. arabaticum* Z-7288^T^

The cells of *A. arabaticum* are bent rods, motile by one to two subterminal flagella (Table 1) [2]. The flagella are stated in the original description [2], though they are not visible in our study (Figure 1). The cells are single, in pairs or form short chains, being 0.7-1 µm in diameter and 1-5 µm in length [2]. Other typical cell aggregates are palisades and ribbons, which are formed by adhesion of cells having intimate contact (Figure 1) [2]. The multiplication is by binary fission. The outer membrane is typical of a Gram-negative organism [2]. Growth is completely inhibited by 100 µM/ml streptomycin, benzylpenicillin, bacitracin, erythromycin, gentamycin, kanamycin, vancomycin or tetracyclin [2]. Strain Z-7288^T^ is obligately anaerobic, tolerating up to 12 mM H_2_S. Neither O_2_, S_2_O_3_^2-^, SO_4_^2-^, nor  S^0^ can serve as electron acceptors. Strain Z-7288^T^ requires a salt concentration of 10-25% NaCl, the optimum is 15-18% NaCl [2]. The optimal pH is between 7.6 and 8.0 [2].

**Table 1:** Classification and general features of *A. arabaticum* Z-7288^T^ according to the MIGS recommendations [4]

**MIGS ID**	**Property**	**Term**	**Evidence code**
	Current classification	Domain *Bacteria*	TAS [5]
		Phylum *Firmicutes*	TAS [6,7]
		Class *Clostridia*	TAS [8,9]
		Order *Halanaerobiales*	TAS [10-12]
		Family *Halobacteroidaceae*	TAS [11,12]
		Genus *Acetohalobium*	TAS [1,13]
		Species *Acetohalobium arabaticum*	TAS [1,13]
		Type strain Z-7288	TAS [2]
	Gram stain	negative	TAS [2]
	Cell shape	bent rod	TAS [2]
	Motility	motile, subterminal flagella	TAS [2]
	Sporulation	unknown; not observed	NAS
	Temperature range	max. 47°C	TAS [2]
	Optimum temperature	28-40°C	TAS [2]
	Salinity	10-25% (optimal 15-18%) NaCl	TAS [2]
MIGS-22	Oxygen requirement	anaerobic	TAS [2]
	Carbon source	CO, CO_2_, TMA, betaine, lactate, pyruvate	TAS [2]
	Energy source	chemolithoautotroph, methylotroph, organotroph	TAS [2]
MIGS-6	Habitat	lagoon	TAS [2]
MIGS-15	Biotic relationship	free-living	TAS [2]
MIGS-14	Pathogenicity	not reported	
	Biosafety level	1	TAS [14]
	Isolation	lagoon	TAS [2]
MIGS-4	Geographic location	Arabat Spit, Ukraine	TAS [2]
MIGS-5	Sample collection time	1990 or before	TAS [2]
MIGS-4.1 MIGS-4.2	Latitude Longitude	46.26 34.86	NAS
MIGS-4.3	Depth	unknown	
MIGS-4.4	Altitude	about 15 m	NAS

Evidence codes - IDA: Inferred from Direct Assay (first time in publication); TAS: Traceable Author Statement (i.e., a direct report exists in the literature); NAS: Non-traceable Author Statement (i.e., not directly observed for the living, isolated sample, but based on a generally accepted property for the species, or anecdotal evidence). These evidence codes are from of the Gene Ontology project [15]. If the evidence code is IDA, then the property was directly observed by one of the authors or an expert mentioned in the acknowledgements.

*A. arabaticum* exhibits three modes of nutrition [2]: It is chemolithoautotrophic using H_2_ together with CO_2_ or CO; it is methylotrophic using trimethylamine (TMA); and it is organotrophic using betaine, lactate, pyruvate or histidine. Carbohydrates are not utilized. No growth occurs on methanol, monomethylamine (MMA), dimethylamine (DMA), dimethylglycine, choline or sarcosine [2]. When grown on TMA, an equimolar amount of acetate is formed along with lesser amounts of DMA and MMA [2]. Betaine is degraded mainly to acetate and minor amounts of methylamines [2].

Carbonic anhydrase (CA; carbonate hydrolyase, EC 4.2.1.1) has been studied in strain Z-7288^T^ and in other acetogenic bacteria [16]. This zinc-containing enzyme is found in animals, plants, bacteria and archaea and catalyzes the following reaction: CO_2_ + H_2_O ↔ HCO_3_^-^ and H^+^ [16]. Further biochemical details of CA are described elsewhere [16]. Strain Z-7288^T^ displayed CA activities similar to those of other CA-containing bacteria [16-18]. With lactate as cultivation substrate the specific activity of CA in strain Z-7288^T^ has been determined to be 2.1± 0.4 units per mg or protein [16]. It has been suggested that one physiological function for CA in acetogens is to increase intracellular CO_2_ levels [16].

The 16S rRNA genes of the other type strains in the family *Halobacteroidaceae* share between 85.9% (*Orenia sivashensis* [19]) and 95.1% (*Sporohalobacter lortetii* [20]) sequence identity with strain Z-7288^T^ [21]. Uncultured clone sequences from environmental samples and metagenomic surveys do not surpass 84-86% sequence similarity to the 16S rRNA gene sequence of strain Z-7288^T^, indicating a lack of further members of the genus *Acetohalobium* in the habitats screened thus far (status June 2010).

**Figure 2:**
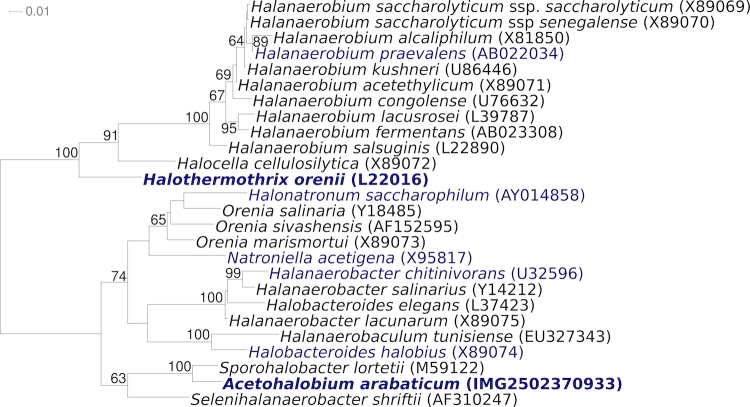
Phylogenetic tree highlighting the position of *A. arabaticum* Z-7288^T^ relative to the type strains of the other genera within the order *Halanaerobiales*. The trees were inferred from 1,308 aligned characters [22,23] of the 16S rRNA gene sequence under the maximum likelihood criterion [24] and rooted in accordance with the current taxonomy [25]. The branches are scaled in terms of the expected number of substitutions per site. Numbers above branches are support values from 300 bootstrap replicates [26] if larger than 60%. Lineages with type strain genome sequencing projects registered in GOLD [27] are shown in blue, published genomes [28] in bold.

Figure 2 shows the phylogenetic neighborhood of *A. arabaticum* Z-7288^T^ in a 16S rRNA based tree. The sequences of the five 16S rRNA gene copies in the genome of *Acetohalobium arabaticum* Z-7288^T^ differ from each other by up to one nucleotide, and differ by up to three nucleotides from the previously published 16S rRNA sequence generated from DSM 5501 (X89077).

### Chemotaxonomy

No chemotaxonomic data are currently available for the genus *Acetohalobium*. 

## Genome sequencing and annotation

### Genome project history

This organism was selected for sequencing on the basis of its phylogenetic position [29], and is part of the *** G****enomic* *** E****ncyclopedia of* *** B****acteria and* *** A****rchaea * project [30]. The genome project is deposited in the Genome OnLine Database [27] and the complete genome sequence is deposited in GenBank. Sequencing, finishing and annotation were performed by the DOE Joint Genome Institute (JGI). A summary of the project information is shown in Table 2.

**Table 2:** Genome sequencing project information

**MIGS ID**	**Property**	**Term**
MIGS-31	Finishing quality	Finished
MIGS-28	Libraries used	Two genomic libraries: 454 pyrosequence standard and paired ended 10kb library
MIGS-29	Sequencing platforms	454 GS Titanium, Illumina GAii
MIGS-31.2	Sequencing coverage	98.4× pyrosequence
MIGS-30	Assemblers	Newbler version 2.0.00.20- PostRelease-11-05-2008-gcc-3.4.6, phrap, Velvet
MIGS-32	Gene calling method	Prodigal 1.4, GenePRIMP
	INSDC ID	CP002105
	Genbank Date of Release	August 9, 2010
	GOLD ID	Gc01329
	NCBI project ID	32769
	Database: IMG-GEBA	2502171194
MIGS-13	Source material identifier	DSM 5501
	Project relevance	Tree of Life, GEBA

### Growth conditions and DNA isolation

*A. arabaticum* Z-7288^T^, DSM 5501, was grown anaerobically in DSMZ medium 494 (*Acetohalobium* medium) [31] at 37°C. DNA was isolated from 0.5-1 g of cell paste using MasterPure Gram Positive DNA Purification Kit (Epicentre MGP04100). Two µl lysozyme and five µl mutanolysin were added to the standard lysis solution for 40min at 37°C followed by 1 hour incubation on ice after the MPC-step.

### Genome sequencing and assembly

The genome of *A. arabaticum* Z-7288^T^ was sequenced at using a combination of Illumina and 454 technologies. An Illumina GAii shotgun library with reads of 483 Mb a 454 Titanium draft library with average read length of 341 bases, and a paired end 454 library with average insert size of 10 kb were generated for this genome. All general aspects of library construction and sequencing can be found at http://www.jgi.doe.gov/. Illumina sequencing data was assembled with VELVET and the consensus sequences were shredded into 1.5 kb overlapped fake reads and assembled together with the 454 data. Draft assemblies were based on 241 Mb 454 draft data, and 454 paired end data. Newbler parameters are -consed -a 50 -l 350 -g -m -ml 20. The initial assembly contained 72 contigs in one scaffold. The initial 454 assembly was converted into a phrap assembly by making fake reads from the consensus, collecting the read pairs in the 454 paired end library. The Phred/Phrap/Consed software package (www.phrap.com) was used for sequence assembly and quality assessment in the following finishing process [32]. After the shotgun stage, reads were assembled with parallel phrap (High Performance Software, LLC). Possible mis-assemblies were corrected with gapResolution (http://www.jgi.doe.gov/), Dupfinisher [32], or sequencing cloned bridging PCR fragments with subcloning or transposon bombing (Epicentre Biotechnologies, Madison, WI). Gaps between contigs were closed by editing in Consed, by PCR and by Bubble PCR primer walks (J.-F. Cheng, unpublished). A total of 292 additional reactions were necessary to close gaps and to raise the quality of the finished sequence. Illumina reads were used to improve the final consensus quality using an in-house developed tool (the Polisher [33], ). The completed genome sequences have an error rate of less than 1 in 100,000 bp.

### Genome annotation

Genes were identified using Prodigal [34] as part of the Oak Ridge National Laboratory genome annotation pipeline, followed by a round of manual curation using the JGI GenePRIMPpipeline [35]. The predicted CDSs were translated and used to search the National Center for Biotechnology Information (NCBI) nonredundant database, UniProt, TIGRFam, Pfam, PRIAM, KEGG, COG, and InterPro databases. Additional gene prediction analysis and functional annotation was performed within the IntegratedMicrobialGenomes-ExpertReview (IMG-ER) platform [36].

## Genome properties

**Figure 3:**
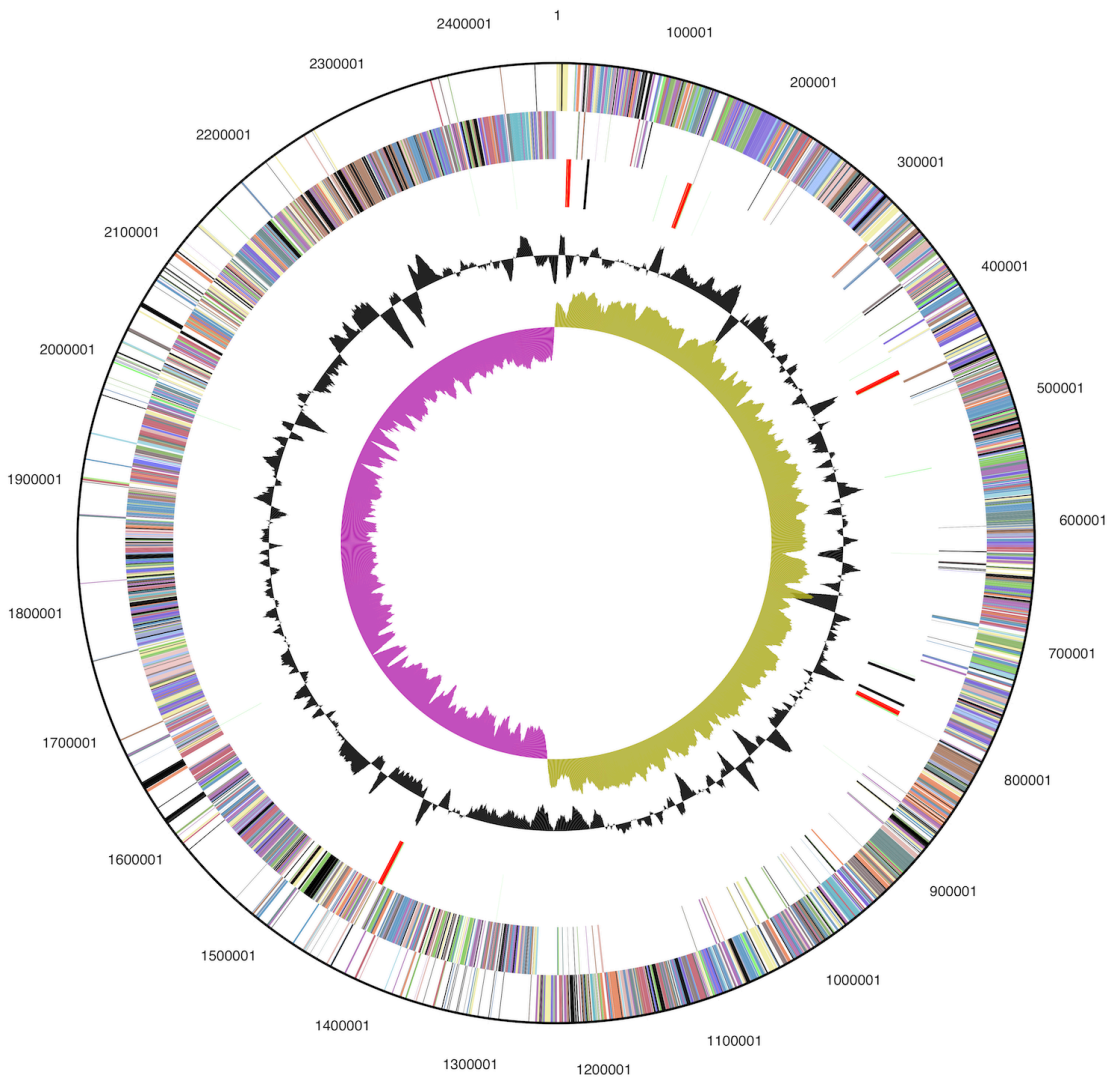
Graphical circular map of the genome. From outside to the center: Genes on forward strand (color by COG categories), Genes on reverse strand (color by COG categories), RNA genes (tRNAs green, rRNAs red, other RNAs black), GC content, GC skew.

The genome consists of a 2,469,596 bp long chromosome with a 36.6% GC content (Table 3 and Figure 3). Of the 2,443 genes predicted, 2,353 were protein-coding genes, and 90 RNAs; Seventy-one pseudogenes were also identified. The majority of the protein-coding genes (76.4%) were assigned a putative function while the remaining ones were annotated as hypothetical proteins. The distribution of genes into COGs functional categories is presented in Table 4.

**Table 3:** Genome Statistics

**Attribute**	**Value**	**% of Total**
Genome size (bp)	2,469,596	100.00%
DNA coding region (bp)	2,147,537	86.96%
DNA G+C content (bp)	904,645	36.63%
Number of replicons	1	
Extrachromosomal elements	0	
Total genes	2,443	100.00%
RNA genes	90	3.67%
rRNA operons	5	
Protein-coding genes	2,353	96.33%
Pseudo genes	71	3.30%
Genes with function prediction	1,873	76.36%
Genes in paralog clusters	505	20.58%
Genes assigned to COGs	1,861	75.87%
Genes assigned Pfam domains	2,022	82.43%
Genes with signal peptides	378	15.41%
Genes with transmembrane helices	303	12.35%
CRISPR repeats	1	

**Table 4:** Number of genes associated with the general COG functional categories

**Code**	**value**	**%age**	**Description**
J	133	6.5	Translation, ribosomal structure and biogenesis
A	0	0.0	RNA processing and modification
K	119	5.8	Transcription
L	139	6.8	Replication, recombination and repair
B	2	0.1	Chromatin structure and dynamics
D	26	1.3	Cell cycle control, cell division, chromosome partitioning
Y	0	0.0	Nuclear structure
V	21	1.0	Defense mechanisms
T	88	4.3	Signal transduction mechanisms
M	123	6.0	Cell wall/membrane/envelope biogenesis
N	66	3.2	Cell motility
Z	0	0.0	Cytoskeleton
W	0	0.0	Extracellular structures
U	56	2.8	Intracellular trafficking, secretion, and vesicular transport
O	85	4.2	Posttranslational modification, protein turnover, chaperones
C	150	7.4	Energy production and conversion
G	62	3.0	Carbohydrate transport and metabolism
E	187	9.2	Amino acid transport and metabolism
F	61	30	Nucleotide transport and metabolism
H	144	7.1	Coenzyme transport and metabolism
I	43	2.1	Lipid transport and metabolism
P	106	5.2	Inorganic ion transport and metabolism
Q	19	0.9	Secondary metabolites biosynthesis, transport and catabolism
R	224	11.0	General function prediction only
S	183	9.0	Function unknown
-	582	24.1	Not in COGs
